# Accuracy of breast density assessment using artificial intelligence by convolutional neural network for carriers of UGT1A1 polymorphisms with Gilbert's Syndrome – a pilot study

**DOI:** 10.1016/j.clinsp.2026.101038

**Published:** 2026-07-09

**Authors:** Jonathan Yugo Maesaka, Bruna Salani Mota, Carlos Shimizu, Bruno Aragão Rocha, Luis Gustavo Rocha Vianna, Giovanni Guido Cerri, José Roberto Filassi, Gustavo Arantes Rosa Maciel, José Maria Soares-Jr, Suzane Kioko Ono, Edmund Chada Baracat

**Affiliations:** a Discipline of Gynecology, Department of Obstetrics and Gynecology, Faculdade de Medicina, Universidade de São Paulo (FMUSP), São Paulo, SP, Brazil; b Department of Radiology, Faculdade de Medicina, Universidade de São Paulo (FMUSP), São Paulo, SP, Brazil; c Instituto de Matemática e Estatística, Universidade de São Paulo (USP), São Paulo, SP, Brazil; d Department of Gastroenterology, Faculdade de Medicina, Universidade de São Paulo (FMUSP), São Paulo, SP, Brazil

**Keywords:** Gilbert disease, Estrogens/metabolism, Breast neoplasms, Breast density

## Abstract

**Introduction:**

*:* Gilbert's Syndrome (GS), an indirect hyperbilirubinemia resulting from the reduced hepatic bilirubin-glucuronyltransferase enzyme activity, influences estrogen metabolism. Polymorphisms in the UGT gene, such as those associated with GS, can alter estrogen clearance, impacting hormonal exposure and, consequently, breast density, an independent risk factor for breast cancer. Furthermore, the accumulation of estrogenic metabolites like 4-OH-estrogens, recognized as carcinogenic agents, suggests a potential increased risk of breast cancer in GS patients. Given this complex interrelationship, this pilot study aimed to evaluate the accuracy of a Convolutional Neural Network (CNN) in determining breast density in postmenopausal women with GS, seeking to optimize their follow-up.

**Methods:**

The authors conducted a prospective, cross-sectional study involving 21 patients, divided into GS and genotyped wild-type control groups. The CNN's performance was compared against radiologists' assessments.

**Results:**

For dense breasts in the GS group, the CNN showed 55.6% sensitivity, 100.0% specificity, and 77.8% accuracy. Kappa agreement was 0.381 for the GS group and 0.700 for controls (fair and good agreement, respectively). Specificity was the most representative diagnostic parameter for dense breasts.

**Conclusion:**

This pilot study provides preliminary evidence suggesting that the CNN's sensitivity in breast density assessment in women with GS may be low, while specificity seems to be high. These findings suggest the need for further validation of artificial intelligence algorithms in specific populations, such as those with GS, and that conclusions should be interpreted with caution due to the small sample size and wide confidence intervals.

## Highlights

• CNN breast density assessment may show variable accuracy in Gilbert's Syndrome.
• AI algorithm validation is crucial for specific metabolic populations.
• First study on AI mammographic density in Gilbert's syndrome patients.
• UGT1A1 polymorphisms impact estrogen metabolism and breast density.

## Introduction

Gilbert's Syndrome is described as an indirect hyperbilirubinemia that occurs in the absence of liver diseases or hemolytic conditions^[^^1^ Intermittent jaundice is its main clinical manifestation. The increase in serum bilirubin levels in GS patients is due to the reduction of hepatic bilirubin-glucuronyltransferase enzyme activity to about 20% of normal^[^^2^ In 1995, the presence of the polymorphism in UGT1A1 was identified as a genotype associated with the clinical manifestation of GS^[^^3^ It is estimated that between 3% and 10% of the population has GS^[^^4^ It is more prevalent in males, with a 3:1 ratio^[^^5^

Clinical evidence relates the diagnosis of GS to modifications in drug and medication metabolism processes,^6^^,^^7^ and to its potential association with the genesis of various diseases, such as diabetes^8^ and coronary artery disease^[^^9^^,^^10^ Some authors refer to an association of GS with cancer^[^^11^^,^^12^

It is known that changes in the glucuronidation pathway occurring in GS would lead to alterations in estrogen metabolism, promoting the accumulation of metabolites that participate in cell proliferation pathways mediated by this hormone^[^^13^ These intermediate metabolites would exert their carcinogenic effect by forming superoxide radicals and other free radicals that damage deoxyribonucleic acid. Furthermore, they would have a direct action on stimulating and inhibiting estrogen receptor signaling pathways, associated with proliferation and apoptosis, phenomena linked to mammary carcinogenesis^[^^14^^,^^15^

Mammography is the gold standard for breast cancer screening. Its sensitivity is influenced by breast density^[^^16^ Breast density is known to be associated with hormonal action, especially estrogen and progesterone. In 2011, Astolfi et al. suggested that GS could be a risk factor for breast cancer due to alterations in estrogen metabolism^[^^17^ As the frequency of this syndrome is low, there is difficulty in establishing the relationship between GS and breast cancer.

Given the incorporation of computer-assisted technologies for evaluating mammographic images, the analysis of potential applications and limitations in specific populations is part of the questions raised by specialists, as such technologies are trained on large general databases^[^^18^

The documented influence of Gilbert's Syndrome on estrogen metabolism and its potential impact on breast density leads to a critical hypothesis: the underlying metabolic milieu in GS patients may result in subtle, yet qualitatively distinct, structural or textural characteristics within dense breast tissue. These unique features, which might not be readily apparent through conventional visual assessment, could inherently differ from the breast tissue patterns typically encountered in the general population. Consequently, a CNN trained predominantly on large databases derived from general populations might not have learned to adequately recognize or interpret these specific qualitative differences in GS patients. This potential mismatch between the learned patterns of the AI model and the distinct biological realities of this specific metabolic subgroup underscores the imperative for rigorous validation of such algorithms in diverse and genetically defined patient cohorts.

This pilot study aimed to evaluate the performance of a CNN algorithm in classifying mammographic density in postmenopausal women with Gilbert’s syndrome, comparing it with genotyped controls.

## Methods

### Study design and participants

This was an exploratory cross-sectional study conducted at the Mastology Service, Gynecology Division, Hospital das Clínicas of the University of São Paulo Medical School, between September 2018 and September 2021. This study was designed and reported in accordance with the Strengthening the Reporting of Observational Studies in Epidemiology (STROBE) Statement. Twenty-one postmenopausal women, aged between 40- and 70-years were included, divided into two groups based on UGT1A1×28 genotyping: Gilbert's Syndrome group and wild-type control group. Patients with HIV or liver cirrhosis were excluded.

### Genotyping

DNA extraction was performed from peripheral blood. The promoter region of the UGT1A1 gene was amplified by PCR and sequenced using an ABI377 sequencer. TA polymorphism analysis was performed using the Bioedit Sequence Alignment Editor program.

### Mammographic evaluation

All participants underwent bilateral digital mammography in craniocaudal and mediolateral oblique views. Mammographic density was evaluated by two methods:

1. Radiological assessment: A Na experienced radiologist classified density according to ACR BI-RADS criteria into four categories: A, B, C, and D (A ‒ Almost entirely fatty; B ‒ Scattered areas of fibroglandular density; C ‒ Heterogeneously dense; D ‒ Extremely dense).
2. CNN assessment: An algorithm previously published by Wu et al., trained on a database of 200,000 mammograms, was used. DICOM images were converted to PNG format, resized to 2000 × 2600 pixels, with horizontal mirroring of right incidences for standardization. The algorithm automatically classified density into the same four BI-RADS categories.

### AI model description

The artificial intelligence assessment in this study utilized a Convolutional Neural Network algorithm initially developed and described by Wu et al. This model is a multi-column deep CNN architecture designed specifically for breast density classification[19] (Wu et al., 2018).

The network's architecture integrates features from the four standard mammography views (left cranio-caudal, right cranio-caudal, left mediolateral oblique, and right mediolateral oblique). Each view is processed through a series of convolutional and pooling layers. The features extracted from these individual view processing streams are then concatenated and fed into a fully connected layer with 1024 hidden units before a final classification layer[19] (Wu et al., 2018).

The model was extensively trained on a large and diverse clinically realistic dataset comprising over 200,000 screening mammography exams. In this initial dataset, there was no clinical information on comorbidities, nor specific hormonal conditions like GS. Each exam typically included at least the four standard views. The breast density labels for training were automatically extracted from the textual radiological reports associated with these exams, classifying images into the four BI-RADS density categories.

For inference on the study images, the original DICOM mammograms were converted to PNG format and uniformly resized to 2000 × 2600 pixels. To ensure consistency in input orientation, right-sided mammographic views were horizontally mirrored. None of these conversions impacted the original evaluation of the CNN. The pre-trained CNN algorithm then received these four processed images as input and directly classified the breast density into one of the four BI-RADS categories. This direct four-way classification output was subsequently dichotomized into “dense” and “non-dense” for the primary analysis in the present study, as detailed in the Statistical Analysis section.

### Statistical analysis

Data were collected and managed using the REDCap platform^[^^20^ For the main analysis, density categories were dichotomized into “dense” and “non-dense”. Agreement between the radiologist and CNN was assessed using Cohen's kappa coefficient. Sensitivity, specificity, positive and negative predictive values were calculated using radiological assessment as the reference standard, expressed as percentages with 95% confidence intervals calculated by the exact Clopper-Pearson method. Likelihood ratios and their confidence intervals were calculated by the Log method. Qualitative variables were compared between groups using the chi-squared test. The Kruskal-Wallis test was used to evaluate the distribution of continuous variables between groups. All tests considered a two-sided significance level ⍺ of 0.05 and a 95% confidence interval. Analyses were performed using IBM SPSS v.25 and Microsoft Excel 2010.

### Ethical aspects

The study has been carried out in accordance with the Declaration of Helsinki, and was approved by the institutional Research Ethics Committee (16,291/2017). All participants signed an informed consent form.

## Results

Twenty-one participants were included in the study, 12 in the GS group and 9 in the Control group. The mean age of the patients was 61 years. Table 1 presents the clinical characteristics of the patients.

**Table 1:** Clinical characteristics of patients, according to inclusion group.

	Gilbert	Control wild type	p-value
**Mean age**	61	61	0.160
**Mean BMI**	28.1	28.2	0.730
**Hypertension (n)**	5	6	0.699
**Diabetes (n)**	4	3	0.663
**Dyslipidemia (n)**	2	3	0.622
**Hypothyroidism (n)**	2	3	0.648
**Breast cancer (n)**	1	0	1.000
**Hepatitis C (n)**	5	1	0.403
**Thyroidopathy (n)**	3	3	0.663
**Familial History Breast Cancer (n)**	3	6	0.225
**Familial History Ovarian Cancer (n)**	0	2	0.067
**Familial History Other cancers (n)**	8	4	0.058
**Gail Index 5-years mean (±SD)**	1.89	1.02	0.286
**Gail Index lifetime mean (±SD)**	8.15	6.06	0.094

All patients were evaluated for breast cancer risk using the Gail index. The Gail index is a widely used tool for assessing breast cancer risk^[^^21^ One patient was excluded from the analysis in the GS group due to a personal history of breast cancer. The groups were homogeneous. Breast density classification by CNN also showed homogeneity (*p* = 0.255 and *p* = 0.884), as observed in Table 2.

**Table 2:** Breast densities evaluated by radiologists and CNN in GS and wild-type control groups, and agreement coefficient of radiologist vs. CNN assessments.

	Gilbert		Wild-type control		p-value
	n	%	n	%	
**Radiologist assessment**					
Dense breasts	7	58.3%	3	33.3%	0.255
Non-dense breasts	5	41.7%	6	66.7%	
**CNN**					
Dense breasts	4	33.3%	3	33.3%	0.884
Non-dense breasts	8	66.7%	6	66.7%	
**Agreement coefficient**					
Radiologist vs. CNN					
Kappa		0.381		0.700	
p-value		0.037		<0.001	

In the stratified analysis according to the investigation groups, the highest kappa correlation coefficient was for the Radiologist vs. CNN comparison in the wild-type control group, with 0.700 and *p* < 0.001.

The diagnostic estimates of the CNN in assessing breast density when compared to radiologist analysis in the four different categories were evaluated. It was observed that for dense breasts, sensitivity was lower in the Gilbert group compared to the wild-type control group, whose sensitivity and specificity were 100%. In patients with dense breasts, specificity was the most representative diagnostic parameter among those evaluated, while accuracy was a good parameter for lipo-substituted breasts (Table S1 ‒ Supplementary Material). The confidence intervals for nearly all diagnostic parameters (e.g., sensitivity of 57.1% with a 95% CI of 18.4%‒90.1% in the GS group for dense breasts) are wide, which reflects the preliminary nature of the findings and the uncertainty introduced by the small sample size.

The data presented in Table 3 detail the diagnostic performance of the CNN in classifying breasts as dense or non-dense compared to radiological assessment. An overall sensitivity of 50.0% (95% CI 32.4%‒67.6%) was observed for the detection of dense breasts, though it is crucial to note the wide confidence interval reflecting considerable statistical uncertainty. Stratifying by group, sensitivity in the Gilbert's Syndrome group was 55.6% (95% CI 21.2%‒86.3%), while in the wild-type control group it was 80.0% (95% CI 28.4%‒99.5%). These estimates, particularly for the Gilbert's Syndrome group, are highly unstable due to the extremely wide confidence intervals, providing only preliminary and highly suggestive evidence of a variable ability of the algorithm to correctly identify dense breasts among these specific populations. Therefore, these findings must be interpreted with extreme caution and are hypothesis-generating rather than definitive. In contrast, specificity was high, reaching 95.8% (95% CI 78.9%‒99.9%) in the total sample, and 100.0% in both the Gilbert and control groups, a suggestive trend that the CNN may be effective in correctly classifying non-dense breasts, though the preliminary nature of these results still necessitates further validation with larger cohorts.

**Table 3:** Diagnostic estimates of the CNN in assessing dense vs. non-dense breasts, compared to radiologist.

	Total		Gilbert		Wild-type control	
	**Estimate**	**95%CI**	**Estimate**	**95%CI**	**Estimate**	**95%CI**
**Dense vs. non-dense**						
Sensitivity	50.0%	32.4% ‒ 67.6%	55.6%	21.2% ‒ 86.3%	80.0%	28.4% ‒ 99.5%
Specificity	95.8%	78.9% ‒ 99.9%	100.0%	47.8% ‒ 100.0%	100.0%	54.1% ‒ 100.0%
Positive likelihood ratio	12	1.71 ‒ 84.2	‒	‒	‒	‒
Negative likelihood ratio	0.522	0.369 ‒ 0.738	0.444	0.214 ‒ 0.923	0.200	0.346 ‒ 1.150
Positive predictive value	94.4%	72.7% ‒ 99.9%	100.0%	47.8% ‒ 100.0%	100.0%	39.8% ‒ 100.0%
Negative predictive value	57.5%	40.9% ‒ 73.0%	55.6%	21.2% ‒ 86.3%	85.7%	42.1% ‒ 99.6%
Accuracy	72.9%	78.2% ‒ 96.7%	77.8%	60.6% ‒ 95.0%	77,78%	39.9% ‒ 97,2%

CI, Confidence Interval.

## Discussion

This study represents the first investigation into the performance of artificial intelligence algorithms in mammographic density analysis in postmenopausal women with Gilbert's syndrome, identifying reduced agreement between CNN and radiological assessment in this specific population, suggesting that algorithms developed and trained in general populations may have limitations when applied to subgroups with specific hormonal and metabolic characteristics.

Although convolutional neural networks have demonstrated high potential to assist mammographic interpretation in broad population studies,^22^ these results indicate that their accuracy may be compromised in particular clinical conditions such as GS. This discrepancy raises fundamental questions about the generalization of AI tools in medicine, particularly when algorithms are developed using extensive databases that may not adequately capture the phenotypic diversity of subpopulations with specific hormonal and metabolic alterations^[^^23^ The observed variation in performance suggests that unique tissue characteristics, possibly related to altered estrogen metabolism in GS, may not be adequately represented in the recognition patterns learned by the algorithm during its training.

The present findings of a higher prevalence of dense breasts in the GS group partially align with the results of Haiman et al., who demonstrated an association between the UGT1A1 TA7/7 genotype and higher mammographic density in postmenopausal women^[^^24^ The magnitude of this difference in the present study, although not statistically significant, suggests a similar trend.

There are no previous studies evaluating CNN performance specifically in GS. Wu et al. reported high algorithm accuracy in the general population but did not stratify results by hormonal and metabolic conditions^[^^19^ Machine learning algorithms, including CNNs, are often trained on large general databases. However, the application of these models in rare populations or with specific clinical conditions like GS can be challenging due to overfitting or a lack of diversity in training data^[^^25^ Overfitting occurs when a model is excessively calibrated to a specific dataset, resulting in lower reproducibility of its performance in different populations and contexts^[^^25^^,^^26^ The ability of an AI model to generalize to different patient data, demographic characteristics, and imaging equipment is crucial for its clinical and ethical effectiveness. This hypothesis is consistent with emerging literature on the need for AI validation in diverse populations^[^^27^^,^^28^

This study is pioneering in specifically investigating the application of AI in a population with a genetically documented metabolic alteration. The genotypic confirmation of all participants represents important methodological rigor, eliminating classifications based solely on clinical criteria. The use of a previously validated and published CNN algorithm, with a standardized image processing methodology, strengthens reproducibility. Additionally, the comparative evaluation with a genotyped control group allows for the isolation of the specific effect of GS.

### Limitations of the study

This is a pilot study with a small sample size, which impacts statistical power and the ability to draw definitive conclusions, increasing the type II error rate. This limitation means that the study may not have had sufficient power to detect clinically relevant differences or associations, if they exist. The wide confidence intervals observed reflect this statistical fragility and prevent definitive conclusions about the real impact of GS on CNN performance. The authors recognize that this sample limitation is partially inherent to the study of low-prevalence conditions like GS. To minimize biases, the authors used confirmatory genotyping of all participants and rigorous standardization of imaging procedures. However, previous validation of the CNN algorithm in other populations and the use of a larger and more diverse sample would robustly consolidate the results obtained here. It is important to note that the absence of specific metabolic or hormonal condition data in the training set may limit the model's ability to recognize tissue patterns unique to populations with conditions like Gilbert's Syndrome.

Another significant limitation of this study, coupled with the small sample size, is the reliance on a single radiologist for breast density assessment. While this was an experienced radiologist, it is widely recognized that visual assessment of breast density carries inherent inter-observer variability, even among experts. Consequently, the reported kappa values and other diagnostic performance metrics in this study are relative to this single reader's interpretation and cannot be considered an absolute “gold standard”. The absence of a second reader or a consensus panel precludes inter-observer agreement analysis and may have introduced assessment bias, further emphasizing the preliminary nature of these findings and the need for future validations with more robust radiological evaluations.

Additionally, the analysis of Table 1 reveals certain clinical differences between the groups that warrant attention as potential confounding factors. Notably, the GS group exhibited a considerably higher prevalence of Hepatitis C (5 vs. 1 case in the control group) and a trend towards a greater family history of other cancers (though not statistically significant due to low statistical power). While the authors aimed to minimize biases through confirmatory genotyping, these differences could have influenced the systemic and hormonal metabolism of patients in the GS group, and consequently, the characteristics of breast density and, therefore, the CNN's performance. The inability to statistically adjust for these potential confounding factors due to the limited sample size means that the observed variations in CNN performance cannot be attributed solely to the UGT1A1 polymorphism. Future studies with larger samples should stratify or adjust for these variables to more precisely isolate the effect of Gilbert's Syndrome.

Despite these limitations, this remains the first specific study evaluating mammographic density through convolutional neural networks in the Gilbert's syndrome population, providing important preliminary data to guide future investigations with more robust designs.

### Conclusions and future perspectives

The current results are preliminary and do not justify immediate changes in clinical practice. However, they are alert to the need for caution in the automated interpretation of mammographic density in GS patients until larger studies confirm or refute these findings. Centers using CNN for screening or density assessment should consider the possibility of variable performance in specific populations. If confirmed, these findings would suggest the need for specific validation or adjustment of algorithmic decision thresholds for patients with known hormonal and metabolic alterations.

Future studies should include: larger samples through multicenter collaboration, allowing stratified analyses by specific genotype and hormonal status; evaluation of multiple CNN algorithms to determine if the limitation is algorithm-specific or generalized; analysis of radiomic features that could explain the observed discordance; correlation with serum biomarkers of estrogen metabolism; longitudinal evaluation to determine if the variation in AI performance has a clinical impact on screening outcomes. The authors also recommend including a cost-effectiveness analysis to determine the feasibility of population-specific algorithmic adjustments.

## Conclusions

This pilot study offers initial evidence that CNN algorithms may exhibit variable performance in mammographic density assessment in women with Gilbert's syndrome. The observed trend of lower agreement between CNN and radiological assessment in the GS group suggests that artificial intelligence tools trained on general populations may necessitate specific validation prior to their application in subgroups with particular hormonal and metabolic characteristics. The present findings underscore the need for significantly larger future investigations to determine if algorithmic adjustments are crucial for ensuring greater diagnostic accuracy in this population. Given the preliminary nature of the present results and the wide confidence intervals, automated interpretation of mammographic density in GS patients should be approached with extreme caution until more robust studies confirm or refute these observations. Comprehensive validation of artificial intelligence algorithms across diverse populations is fundamental to ensuring equitable and accurate clinical application of these emerging technologies.

## Funding

This research did not receive any specific grant from funding agencies in the public, commercial, or not-for-profit sectors.

## Data availability

The datasets generated and/or analyzed during the current study are available from the corresponding author upon reasonable request.

## CRediT authorship contribution statement

**Jonathan Yugo Maesaka:** Conceptualization, Data curation, Formal analysis, Investigation, Writing – original draft, Writing – review & editing. **Bruna Salani Mota:** Formal analysis, Writing – review & editing. **Carlos Shimizu:** Formal analysis. **Bruno Aragão Rocha:** Formal analysis. **Luis Gustavo Rocha Vianna:** Formal analysis. **Giovanni Guido Cerri:** Formal analysis. **José Roberto Filassi:** Formal analysis. **Gustavo Arantes Rosa Maciel:** Conceptualization. **José Maria Soares-Jr:** Conceptualization, Formal analysis, Writing – original draft. **Suzane Kioko Ono:** Conceptualization, Formal analysis, Supervision. **Edmund Chada Baracat:** Conceptualization, Supervision, Writing – original draft, Writing – review & editing.

## Declaration of competing interest

The authors declare no conflicts of interest.

## Appendix Supplementary materials

Image, application 1

## Footnotes

Supplementary material associated with this article can be found, in the online version, at doi:10.1016/j.clinsp.2026.101038.

## References

[1] Burchell B., Hume R. (1999). Molecular genetic basis of Gilbert's syndrome. Review. J Gastroenterol Hepatol.

[2] Arias I.M., London I.M. (1957). Bilirubin glucuronide formation in vitro; demonstration of a defect in Gilbert's disease. Science.

[3] Bosma P.J., Chowdhury J.R., Bakker C., Gantla S., de Boer A., Oostra B.A. (1995). The genetic basis of the reduced expression of bilirubin UDP-glucuronosyltransferase 1 in Gilbert's syndrome. N Engl J Med.

[4] Hasegawa Y., Ando Y., Ando M., Hashimoto N., Imaizumi K., Shimokata K. (2006). Pharmacogenetic approach for cancer treatment-tailored medicine in practice. Ann N Y Acad Sci.

[5] Vitek L., Tiribelli C. (2023). Gilbert's syndrome revisited. Review. J Hepatol..

[6] Gil J., Sasiadek M.M. (2012). Gilbert syndrome: the UGT1A1×28 promoter polymorphism as a biomarker of multifactorial diseases and drug metabolism. Review. Biomark Med.

[7] Takano M., Sugiyama T. (2017). UGT1A1 polymorphisms in cancer: impact on irinotecan treatment. Pharmacogenomics Pers Med.

[8] Breimer L.H., Mikhailidis D.P. (2016). Does bilirubin protect against developing diabetes mellitus?. J Diabetes Complicat.

[9] Higashi Y., Maruhashi T., Noma K., Kihara Y. (2014). Oxidative stress and endothelial dysfunction: clinical evidence and therapeutic implications. Trends Cardiovasc Med.

[10] Bulmer A.C., Verkade H.J., Wagner K.H. (2013). Bilirubin and beyond: a review of lipid status in Gilbert's syndrome and its relevance to cardiovascular disease protection. Prog Lipid Res.

[11] Freisling H., Khoei N.S., Viallon V., Wagner K.H. (2020). Gilbert's syndrome, circulating bilirubin and lung cancer: a genetic advantage?. Thorax.

[12] Bugano D.D.G., Conforti-Froes N., Yamaguchi N.H., Baracat E.C. (2008). Genetic polymorphisms, the metabolism of estrogens and breast cancer: a review. Eur J Gynaecol Oncol.

[13] Guillemette C., De Vivo I., Hankinson S.E., Haiman C.A., Spiegelman D., Colditz G.A. (2001). Association of genetic polymorphisms in UGT1A1 with breast cancer and plasma hormone levels. Cancer Epidemiol Biomarkers Prev.

[14] Yager J.D., Davidson N.E. (2006). Mechanisms of disease: estrogen carcinogenesis in breast cancer. Review. N Engl J Med.

[15] Cavalieri E., Stack D., Devanesan P., Todorovic R., Dwivedy I., Higginbotham S. (1997). Molecular origin of cancer: catechol estrogen-3,4-quinones as endogenous tumor initiators. Proc Natl Acad Sci U S A.

[16] Reis Y., Mota B., Mota R., Lacerda M., Costa M., Oliveira A. (2023). Pathological macroscopic evaluation of breast density versus mammographic breast density in breast cancer conserving surgery. Eur J Obstet Gynecol Reprod Biol X.

[17] Astolfi R., Bugano D.D.G., Francisco A., de Souza M., Ono-Nita S.K., Baracat E.C. (2011). Is Gilbert syndrome a new risk factor for breast cancer?. Med Hypotheses.

[18] Branco P., Franco A., de Oliveira A., Martins J., Silva R., Costa M. (2024). Artificial intelligence in mammography: a systematic review of the external validation. Rev Bras Ginecol Obstet.

[19] Wu N., Geras K.J., Shen Y., Su J., Kim S.G., Kim E. (2018). 2018 IEEE International Conference on Acoustics, Speech and Signal Processing (ICASSP). Piscataway (NJ).

[20] Harris P.A., Taylor R., Thielke R., Payne J., Gonzalez N., Conde J.G. (2009). Research electronic data capture (REDCap): a metadata-driven methodology and workflow process for providing translational research informatics support. J Biomed Inform.

[21] Gail M.H., Brinton L.A., Byar D.P., Corle D.K., Green S.B., Schairer C. (1989). Projecting individualized probabilities of developing breast cancer for white females who are being examined annually. J Natl Cancer Inst.

[22] Yoon J., Kim E. (2021). Deep learning-based artificial intelligence for mammography. Korean J Radiol.

[23] Sendak M.P., D'Arcy J., Kashyap S., Gao M., Nichols M., Corey K. (2020). A path for translation of machine learning products into healthcare delivery. EMJ Innov.

[24] Haiman C.A., Hankinson S.E., De Vivo I., Guillemette C., Ishibe N., Hunter D.J. (2003). Polymorphisms in steroid hormone pathway genes and mammographic density. Breast Cancer Res Treat.

[25] Kulkarni V., Gawali M., Kharat A. (2021). Key technology considerations in developing and deploying machine learning models in clinical radiology practice. JMIR Med Inform.

[26] Koh D., Papanikolaou N., Bick U., Khan S., Woznitza N., Mello-Thoms C. (2022). Artificial intelligence and machine learning in cancer imaging. Commun Med.

[27] Hsu W., Hippe D.S., Nakhaei N., Wang C., Abbasi-Sureshjani S., Bedard P.L. (2022). External validation of an ensemble model for automated mammography interpretation by artificial intelligence. JAMA Netw Open.

[28] Huang S., Chaudhari A., Langlotz C., Shah N., Yeung S., Lungren M. (2022). Developing medical imaging AI for emerging infectious diseases. Nat Commun.

