# Supplementary material for: Accuracy of breast density assessment using artificial intelligence by convolutional neural network for carriers of UGT1A1 polymorphisms with Gilbert's Syndrome – a pilot study
Source: Clinics (Sao Paulo). 2026 Jul 9;81:101038. doi: 10.1016/j.clinsp.2026.101038 (PMC13380211; doi:10.1016/j.clinsp.2026.101038)
Supplement: Supplementary file 1 [file mmc1.docx]

**CLINICS-D-25-01724**

**Supplementary Material**

**Table S1** Diagnostic estimates of the CNN in assessing breast density compared to radiologist analysis in different categories.

|  | **Total** | | **Gilbert** | | **Wild-type control** | |
| --- | --- | --- | --- | --- | --- | --- |
|  | **Estimate** | **95%CI** | **Estimate** | **95%CI** | **Estimate** | **95%CI** |
| **Extremely dense** |  |  |  |  |  |  |
| Sensitivity | 0.0% | 0.0% ‒ 52.2% | 0.0% | 0.0% ‒ 97.5% | 0.0% | 0.0% ‒ 84.2% |
| Specificity | 100.0% | 92.1% ‒ 100.0% | 100.0% | 71.5% ‒ 100.0% | 100.0% | 59.0% ‒ 100.0% |
| Positive likelihood ratio | ‒ | ‒ | ‒ | ‒ | ‒ | ‒ |
| Negative likelihood ratio | 1 | 1.0 ‒ 1.0 | 1 | 1.0 ‒ 1.0 | 1 | 1.0 ‒ 1.0 |
| Positive predictive value | ‒ | ‒ | ‒ | ‒ | ‒ | ‒ |
| Negative predictive value | 90.0% | 90.0% ‒ 90.0% | 91.7% | 91.7% ‒ 91.7% | 77.8% | 77.8% ‒ 77.8% |
| Accuracy | 90.0% | 78.2% ‒ 96.7% | 91.7% | 61.5% ‒ 99.8% | 77.8% | 39.9% ‒ 97.1% |
| **Heterogeneously dense** |  |  |  |  |  |  |
| Sensitivity | 47.8% | 26.8% ‒ 69.4% | 66.7% | 22.3% ‒ 95.7% | 100.0% | 2.5% ‒ 100.0% |
| Specificity | 82.7% | 64.2% ‒ 94.1% | 100.0% | 54.1% ‒ 100.0% | 75,00% | 34.9% ‒ 96.8% |
| Positive likelihood ratio | 2.77 | 1.12 ‒ 6.85 | ‒ | ‒ | 4 | 1.20 ‒ 13.28 |
| Negative likelihood ratio | 0.63 | 0.41 ‒ 0.96 | 0.33 | 0.11 ‒ 1.03 | 0 | ‒ |
| Positive predictive value | 68.7% | 47.1% ‒ 84.5% | 100.0% | ‒ | 33.3% | 13.1% ‒ 62.4% |
| Negative predictive value | 66.7% | 56.6% ‒ 75.3% | 75.0% | 49.2% ‒ 90.3% | 100.0% | ‒ |
| Accuracy | 67.3% | 52.9% ‒ 79.7% | 83.3% | 51.6% ‒ 97.9% | 77.8% | 39.9% ‒ 97.2% |
| **Scattered areas of fibroglandular density** |  |  |  |  |  |  |
| Sensitivity | 73.3% | 44.9% ‒ 92.2% | 50.0% | 6.7% ‒ 93.2% | 100.0% | 29.2% ‒ 100.0% |
| Specificity | 62.2% | 44.7% ‒ 77.5% | 62.5% | 24.5% ‒ 91.5% | 100.0% | 54.1% ‒ 100.0% |
| Positive likelihood ratio | 1.94 | 1.16 ‒ 3.24 | 1.33 | 0.35 ‒ 5.03 | ‒ | ‒ |
| Negative likelihood ratio | 0.43 | 0.18 ‒ 1.03 | 0.80 | 0.26 ‒ 2.45 | 0 | ‒ |
| Positive predictive value | 44.0% | 31.9% ‒ 56.7% | 40.0% | 15.0% ‒ 71.5% | 100.0% | ‒ |
| Negative predictive value | 85.2% | 70.5% ‒ 93.2% | 71.4% | 44.9% ‒ 88.4% | 100.0% | ‒ |
| Accuracy | 65.4% | 50.9% ‒ 78.0% | 58.3% | 27.7% ‒ 84.8% | 100.0% | 66.3% ‒ 100.0% |
| **Almost entirely fatty** |  |  |  |  |  |  |
| Sensitivity | 88.9% | 51.7% ‒ 99.7% | 100.0% | 2.5% ‒ 100.0% | 100.0% | 29.2% ‒ 100.0% |
| Specificity | 93.0% | 80.9% ‒ 98.5% | 81.8% | 48.2% ‒ 97.7% | 100.0% | 54.1% ‒ 100.0% |
| Positive likelihood ratio | 12.74 | 4.18 ‒ 38.88 | 5.50 | 1.57 ‒ 19.27 | ‒ | ‒ |
| Negative likelihood ratio | 0.12 | 0.02 ‒ 0.76 | 0 | ‒ | 0 | ‒ |
| Positive predictive value | 72.7% | 46.6% ‒ 89.1% | 33.3% | 12.5% ‒ 63.7% | 100.0% | ‒ |
| Negative predictive value | 97.5% | 86.3% ‒ 99.6% | 100.0% | ‒ | 100.0% | ‒ |
| Accuracy | 92.3% | 81.5% ‒ 97.8% | 83.3% | 51.6% ‒ 97.9% | 100.0% | 66.4% ‒ 100.0% |
| **Extremely dense + Heterogeneously dense** |  |  |  |  |  |  |
| Sensitivity | 53.6% | 33.9% ‒ 72.5% | 57.1% | 18.4% ‒ 90.1% | 100.0% | 29.2% ‒ 100.0% |
| Specificity | 95.8% | 78.9% ‒ 99.9% | 100.0% | 47.8% ‒ 100.0% | 100.0% | 54.1% ‒ 100.0% |
| Positive likelihood ratio | 12.86 | 1.83 ‒ 90.32 | ‒ | ‒ | ‒ | ‒ |
| Negative likelihood ratio | 0.48 | 0.32 ‒ 0.73 | 0.43 | 0.18 ‒ 1.01 | 0 | ‒ |
| Positive predictive value | 93.7% | 68.1% ‒ 99.1% | 100.0% | ‒ | 100.0% | ‒ |
| Negative predictive value | 63.9% | 54.1% ‒ 72.6% | 62.5% | 41.5% ‒ 79.7% | 100.0% | ‒ |
| Accuracy | 73.1% | 58.9% ‒ 84.4% | 75.0% | 42.8% ‒ 94.5% | 100.0% | 66.4% ‒ 100.0% |

CI, Confidence Interval.
